# Hsa-miR-155-5p Up-Regulation in Breast Cancer and Its Relevance for Treatment With Poly[ADP-Ribose] Polymerase 1 (PARP-1) Inhibitors

**DOI:** 10.3389/fonc.2020.01415

**Published:** 2020-08-12

**Authors:** Barbara Pasculli, Raffaela Barbano, Andrea Fontana, Tommaso Biagini, Maria Pia Di Viesti, Michelina Rendina, Vanna Maria Valori, Maria Morritti, Sara Bravaccini, Sara Ravaioli, Evaristo Maiello, Paolo Graziano, Roberto Murgo, Massimiliano Copetti, Tommaso Mazza, Vito Michele Fazio, Manel Esteller, Paola Parrella

**Affiliations:** ^1^Fondazione IRCCS Casa Sollievo Della Sofferenza, Laboratorio di Oncologia, San Giovanni Rotondo, Italy; ^2^Fondazione IRCCS Casa Sollievo Della Sofferenza, UO di Biostatistica, San Giovanni Rotondo, Italy; ^3^Fondazione IRCCS Casa Sollievo Della Sofferenza, Laboratory of Bioinformatics Unit, San Giovanni Rotondo, Italy; ^4^Fondazione IRCCS Casa Sollievo Della Sofferenza, UO di Oncologia, San Giovanni Rotondo, Italy; ^5^Istituto Scientifico Romagnolo per lo Studio e la Cura dei Tumori (IRST) IRCCS, Biosciences Laboratory, Meldola, Italy; ^6^Fondazione IRCCS Casa Sollievo Della Sofferenza, UO di Anatomia Patologica, San Giovanni Rotondo, Italy; ^7^Fondazione IRCCS Casa Sollievo Della Sofferenza, UO di Chirurgia Senologica, San Giovanni Rotondo, Italy; ^8^Josep Carreras Leukaemia Research Institute (IJC), Badalona, Spain; ^9^Centro de Investigación Biomédica en Red Cáncer (CIBERONC), Madrid, Spain; ^10^Physiological Sciences Department, School of Medicine and Health Sciences, University of Barcelona, Barcelona, Catalonia, Spain; ^11^Institució Catalana de Recerca i Estudis Avançats (ICREA), Barcelona, Catalonia, Spain

**Keywords:** breast cancer, hsa-miR-155-5p, homologous recombination, PARP-1 inhibitors, Olaparib

## Abstract

miR-155-5p is a well-known oncogenic microRNA, showing frequent overexpression in human malignancies, including breast cancer. Here, we show that high miR-155-5p levels are associated with unfavorable prognostic factors in two independent breast cancer cohorts (CSS cohort, *n* = 283; and TCGA-BRCA dataset, *n* = 1,095). Consistently, miR-155-5p results as differentially expressed in the breast cancer subgroups identified by the surrogate molecular classification in the CSS cohort and the PAM50 classifier in TCGA-BRCA dataset, with the TNBC and *HER2*-amplified tumors carrying the highest levels. Since the analysis of TCGA-BC dataset also demonstrated a significant association between miR-155-5p levels and the presence of mutations in homologous recombination (HR) genes, we hypothesized that miR-155-5p might affect cell response to the PARP-1 inhibitor Olaparib. As expected, miR-155-5p ectopic overexpression followed by Olaparib administration resulted in a greater reduction of cell viability as compared to Olaparib administration alone, suggesting that miR-155-5p might induce a synthetic lethal effect in cancer cells when coupled with PARP-1-inhibition. Overall, our data point to a role of miR-155-5p in homologous recombination deficiency and suggest miR-155-5p might be useful in predicting response to PARP1 inhibitors in the clinical setting.

## Introduction

Breast cancer (BC) is the most frequent cancer among women worldwide, accounting for 13% of all cancer-related deaths (1). Breast cancer is a heterogeneous disease that includes different histological and molecular entities, clinical presentations, and behaviors, which vary in prognosis and response to therapy. To date, Oestrogen Receptor (ER), Progesterone Receptor (PgR), human epidermal growth factor receptor 2 (HER2), and Ki67, together with age, tumor size, histological grade, and lymph node engagement still represent the most reliable markers that provide prognostic information (2). Lately, microarray-based gene expression studies have identified intrinsic breast cancer subtypes of biological and, more importantly, clinical relevance consisting of two oestrogen receptor positive (ER+) subtypes, characterized by a relatively low (luminal A) and high (luminal B) expression of proliferation-related genes, a subtype enriched for HER2-amplified tumors (HER2-enriched), a subtype characterized by the absence of ER, PgR expression and HER2 amplification (Basal-Like) and a subtype ER +/– and negative for, PgR, HER2, claudin 3, claudin 4, claudin 7, and E-cadherin (Claudin-low) (3–6). This information has helped develop risk scores based on differential breast cancer molecular profiles that are currently entering the clinical practice to identify low-risk breast cancer patients who may avoid adjuvant treatment (7). Nevertheless, even these classification systems do not account for all the reported pathological and clinical heterogeneity of breast cancer.

MicroRNAs (miRNAs) are small endogenous non coding RNAs that fine-tune gene expression by post-transcriptional silencing of target mRNAs. Among the plethora of miRNAs that have been linked to human cancers, miR-155-5p (hsa-miR-155-5p) stands out as prominent oncomiR showing frequent overexpression in several hematological and solid tumors (8–12). In breast cancer, miR-155-5p has been found mostly upregulated, and associated with high-grade tumors, advanced stages, and lymph node metastases as well as worse disease-free and overall survival (13–22). At functional level, miR-155-5p validated target genes potentially place miR-155-5p within several cancer-related pathways encompassing cell proliferation, block of differentiation, epithelial-mesenchymal transition (EMT), and even DNA damage repair (DDR) (12, 23–26). However, these relationships between miR-155-5p and breast cancer clinical markers, such as ER and PgR status and tumor subtype, along with its causal role in breast cancer development remain controversial, likely due to limited patient sample sizes and discrepancies among studies in terms of methodologies and experimental models.

Herein, we report the results from the expression analysis of hsa-miR-155-5p we performed in a large cohort of 283 breast cancer patients with a complete median follow-up of 81 months that likely enabled us to define robust relationships between miR-155-5p and clinical parameters. We confirm that miR-155-5p expression is associated with unfavorable prognostic indicators in both our cohort and the TCGA-breast cancer dataset (TCGA-BRCA). We also found that higher miR-155-5p levels correlate with the basal-like subgroup followed by HER2-enriched tumors, whereas lower levels characterize the Luminal A and Luminal B tumors. Moreover, high miR-155-5p expression was found in breast cancer tumors from the TCGA dataset carrying mutations in HR genes. Lastly, we propose that miR-155-5p might play a role in the response to the poly(ADP-ribose) polymerase-1 (PARP-1) inhibitor Olaparib (AZD2281) which have been receiving much attention as promising therapeutic strategy beyond *BRCA-*mutant tumors, such as those with BRCAness and homologous recombination deficiency (HRD).

## Materials and Methods

### Study Design

We evaluated hsa-miR-155-5p expression in a retrospective (prospectively collected) cohort of 283 breast cancer cases with a median follow-up of 81-months (“Casa Sollievo della Sofferenza”, hereafter CSS, cohort). The study was conducted according to the REporting of tumor MARKer Studies (REMARK) guidelines (27). Breast cancer samples were collected at the Breast-Unit, IRCCS “Casa Sollievo della Sofferenza.” Upon receipt from surgery, tissues from the bulk of the tumor were sampled by the pathologist, then snap-frozen in liquid nitrogen and stored at −80°C. For legal reasons, only one tumor specimen (~50–100 mg of frozen tissue in weight) could be collected from each patient. Only women older than 18 years of age in were included in the study. All methods were carried out following the international Helsinki Declaration (7th rev, 2013, EU Directive 2004/23/EC) and Italian (D. Lgs. 30/06/2003, n. 196) regulations for research on patients. The experimental procedures of this study were approved by the Ethical Committee of the IRCCS “Casa Sollievo della Sofferenza” (Prot. N° 140/CE). Prior written informed consent was obtained from all patients in agreement with the experimental protocol approved by the Ethical Committee. All patients received either breast-conserving surgery or total mastectomy, plus sentinel node biopsy, or complete axillary dissection. The post-surgery treatments were performed according to the following guidelines: AIOM (Associazione Italiana Oncologia Medica), St Gallen, National Comprehensive Cancer Network (NCCN) and American Society of Clinical Oncology (ASCO). Progression was defined as evidence of loco-regional (recurrence) and/or distant disease over 4 months from diagnosis and after curative-intent surgical treatment. Clinical data were collected at each of the scheduled follow-up times.

### Clinicopathological Data

Pathological assessment consists of evaluating the histological type, grade and stage. ER, PgR, Ki-67 labeling index, and HER2 status were determined by immunohistochemistry (IHC). Hormone receptor positive BCs, referred to as cases expressing oestrogen (ER) or progesterone (PgR) receptors in ≥1% of neoplastic cells by international guidelines (28), and *HER2-*amplified BCs were established according to standard recommendations (29). Of the 283 patients, 106 (38%) were lymph node negative, and 177 (62%) lymph node positive. Supplemental Table 1 summarizes descriptive statistics for the 283 cases from the CSS cohort analysed in this study. The median age of the study population was 59 years (range, 29–89), and the median tumor size was 2.5 cm (range, 0.5–11.0). Metastases at diagnosis were found in 15 cases whereas among non-metastatic patients (*N* = 268), 55 experienced disease progression (Incidence Rate, IR 3.25 events per 100 PY), and 30 of them (IR 1.65 events per 100 PY) died for the disease.

### Cell Lines, Culture Conditions, and Reagents

*In vitro* assays were performed in a series of four breast cancer cell lines, encompassing the molecular subtypes of TNBC, which were as follows: MDA-MB-231 (claudin-low), MDA-MB-436 (basal-like), MDA-MB-468 (basal-like), and MDA-MB-453 (LAR (Luminal-Androgen-Receptor) subtype). MDA-MB-436 cell line was used as experimental model of *BRCA1-*mutant cells as carrying the pathogenic variant 5396+1G>A in the splice donor site of exon 20 resulting in a BRCA1 truncated protein (30). MDA-MB-436 (HTB-130, lot#63048503, p19), MDA-MB-468 (HTB-132, lot#63226339, p340), and MDA-MB-453 (HTB-131, lot#62959336, p348) cell lines were purchased from ATCC (American Type Culture Collection). MDA-MB-231 (Cat No. 92020424, lot#11D011, p40) cell line was purchased from ECACC (European Collection of Authenticated Cell Cultures). Culture conditions were as follows: MDA-MB-231, MDA-MB-468, and MDA-MB-453 were maintained in MEM w/o L-Gln, 10% FBS, 2 mM L-Gln, 1XNEAA, whereas MDA-MB-436 were maintained in MEM w/o L-Gln 10% FBS, 2 mM L-Gln, 1XNEAA, 5 mM NaP^*^, Human Insulin 10 μg/mL, GSH 16 μg/Ml. All cell lines were grown in a 5% CO_2_ humidified incubator at 37 °C. All cell lines were mycoplasma tested by Hoechst DNA staining and PCR by using N-GARDe Mycoplasma PCR kit (Euroclone).

The PARP-1 inhibitor Olaparib (AZD2281) was kindly provided by AstraZeneca, and dissolved in DMSO as a stock solution of 10 mg/mL.

### RNA Isolation and Quantitative Reverse Transcription Polymerase Chain Reaction (RT-qPCR) Analysis

Total RNA from tissues and cells was isolated according to standard TRIzol^TM^ protocol (Invitrogen, Thermo Fisher Sc). RNA samples from CSS cohort were selected as previously described (31). To assess miR-155-5p expression levels in our cohort of breast cancers, we applied a quantification method with standard curve. Briefly, for both miR-155-5p (ID 002623, Thermo Fisher Sc) and RNU48 (ID 001006, Thermo Fisher Sc) endogenous control, standard curves were constructed by plotting the threshold cycle (Ct) values against logarithm10 of the copy number and fitting by linear least square regression. For each sample, real-time PCR reactions were run in triplicate on ABI PRISM 7900 HT Sequence Detection System (Thermo Fis Sc). For tissue samples, miR-155-5p expression levels were determined as the ratio of the miR-155-5p copy number to the RNU48 copy number and then multiplied by 1000 for easier tabulation [i.e., (hsa-miR-155-5p/RNU48) × 1,000] (31).

### Statistical and Bioinformatics Analyses

Patients' baseline characteristics were reported as median along with interquartile range (IQR, i.e., first-third quartiles) or frequencies and percentages for continuous and categorical variables, respectively. Comparisons between miR-155-5p levels and clinical pathological characteristics were assessed by Pearson correlation with continuous variables and by the two-sample *t*-test (or ANOVA model as appropriate) or Mann–Whitney *U*-test (or Kruskal–Wallis test when appropriate). In case of ANOVA model, pairwise comparisons were assessed from statistical contrasts (defined within the model) and computed *p*-values were adjusted following the step-up Bonferroni method due to Hochberg. In the case of the Kruskal–Wallis test, Dunn's *post-hoc* tests were carried out on each pair of groups and *p*-values were adjusted using the Bonferroni method. The assumption of normality distribution was checked by means of Q–Q plots and Shapiro–Wilks test. As the distribution of miR-155-5p expression levels in the CSS cohort was log-normal, all statistical analyses that involved this cohort were performed on their log-transformed values. Comparisons between distributions of categorical variables were assessed by Chi-Square test. Time-to-event analysis was performed in patients without metastases at diagnosis by univariable proportional hazards Cox regression models. Overall Survival (OS) was defined as the time between the enrollment date and cancer-related death. Progression Free Survival (PFS) was defined as the time between the enrollment date and the tumor progression. Metastasis Free Survival (MFS) was defined as the time between the enrollment date and the development of distant metastases. For patients who did not develop the event of interest, the endpoints (i.e., OS, PFS, and MFS) were defined as the time between the enrollment date and the date of the last available follow-up control. Incidence rates (IR, i.e., mortality and disease progression rates) were reported as number of events per 100 person-years (PY). A two-sided *p* < 0.05 was considered for statistical significance. All statistical analyses were performed using SAS Release 9.4 (SAS Institute, Cary, NC, USA) and plots were produced using R Foundation for Statistical Computing version 3.6. The PRISM software (version 5, GraphPad, Inc.) was used to make graphs and statistical analysis of *in vitro* assays. Single nucleotide variations (missense, frameshift, nonsense, and splicing SNV), copy number variations (CNV), gene, and miRNA expression data and clinical information concerning the TCGA-BRCA cohort, divided into tumor without synchronous metastases and healthy individuals, were retrieved from the NCI Genomic Data Commons (GDC, https://portal.gdc.cancer.gov/) data portal as pre-processed raw files. Germline point mutations in *BRCA1* and *BRCA2* genes were retrieved from cBioPortal (TCGA PanCancer Atlas subset, https://www.cbioportal.org/). Individuals were classified into Prediction Analysis of Microarray 50 (PAM50) subgroups by means of the R/Bioconductor TCGAbiolinks package. Files and data were manipulated using the R Foundation for Statistical Computing version 3.6. Clinicopathological characteristics of these individuals were summarized in Supplemental Table 2. The median age of the study population was 59 years (range, 27–90) and the median follow up was 12 months (IQR 3–35 months). Of the 1,095 patients, 991 (90%) were alive, and 104 (10%) died for the disease.

### Determination of Short-Term Olaparib IC50 (IC50_72 h_) by PrestoBlue^TM^ Viability Reagent

BC cells were seeded at 14,000 cells per well onto 96-well-plates and treated with a series of 2-fold Olaparib serial dilutions ranging from 230 to 1.8 μM, or vehicle (DMSO), in a final volume of 90 μL. For each treatment and vehicle condition, six technical replicates were prepared. Moreover, for each cell line, determination of Olaparib IC50_72h_ was repeated in three independent 96-well-plates at different times. Cell viability was then evaluated after 72 h of treatment by PrestoBlue viability reagent (Thermo Fisher Sc). Briefly, 10 μL of the ready-to-use PrestoBlue Reagent were added directly to the medium. The 96-well-plates were incubated at 37°C for 2 h, and fluorescence was measured on a plate reader (Synergy HT Microplate Reader, BioTek) at 560 nm. Cell viability was calculated as the percentage of cells inhibited by the treatment as measured by the ratio in fluorescence between Olaparib-treated and vehicle-treated (DMSO) cells. The percentage of viable cells was plotted against Log concentrations of Olaparib. Individual data points on dose-response curves are shown as mean ± SD of *n* = 3 biological replicates. The Olaparib IC50_72h_ for BC cell lines, i.e., the concentration of Olaparib causing a 50% reduction in cell viability in a time-interval of 72 h, were as followed: MDA-MB-436 IC50_72h_ 25.8 μM (95%CI: 24.52–27.15), MDA-MB-231 IC50_72h_ 56.8 μM (95%CI: 53.71–59.98), MDA-MB-468 IC50_72h_ 33.81 μM (95%CI: 31.24–36.60), and MDA-MB-453 IC50_72h_ 175 μM (95%CI: 160.8–190.6).

### *In vitro* Transient Transfection of miR-155-5p Mimic/Inhibitor and Olaparib Treatment

To achieve transient overexpression or inhibition of miR-155-5p before Olaparib treatment, BC cells were first seeded onto 96-well-plates at 14,000 cells/well and 6-well-plates at 4.1 × 10^4^ cells/well. Next, BC cell lines were transfected by using 50 nM mirVana miR-155-5p mimic (MC12601, Thermo Fisher Sc.) or mirVana miR-155-5p inhibitor (MH12601, Thermo Fisher Sc.), and correspondent negative controls (mirVana™ miRNA Mimic, Negative Control #1 and mirVana™ miRNA Inhibitor Negative Control #1, respectively, Thermo Fisher Sc.) according to Lipofectamine RNAiMAX forward transfection protocol (Thermo Fisher Sc). For each condition, eight and two technical replicates were prepared in each 96-well and 6-well-plate.

After 24 h from transfection, cell lines were treated with correspondent Olaparib IC50_72h_, except for MDA-MB-453 cell line, which was treated with a lower Olaparib dose (i.e., IC40_72h_, 60 μM) because showing higher sensitivity, in terms of cell tolerance, to liposomes-mediated transfection. Next, cell viability upon miR-155 modulation followed by 72-h exposure to Olaparib was measured by PrestoBlue^TM^ viability reagent as described above.

To evaluate the true effects of miR-155-5p and Olaparib combination on cell viability and rule out those related to vehicle- and transfection procedures, experimental results are presented as comparison between miR-155 overexpressing/downregulated cells and control c(-) cells, treated with Olaparib or vehicle (DMSO). Data are shown as mean ± SD of *n* = 4 biological replicates (Student's *t*-test, ^*^p < 0.05, ^**^p < 0.01; ^***^p < 0.001).

To assess the extent of miR-155-5p overexpression/inhibition by RT-qPCR, adherent cells were harvested by both 96-well-plates and 6-well-plates, and processed for RNA isolation as described above. For each cell line, miR-155-5p levels in mimic/inhibitor-transfected cells were calculated as fold change (2^−ΔΔCT^) to correspondent negative control c(–)-transfected cells.

### Western Blotting Analysis

Whole protein extracts from 6-well-plate seeded-cells were obtained by RIPA buffer (Tris-HCl 10 mM pH 7.5, NaCl 140 mM, Sodium deoxycholate 1%, sodium orthovanadate 1 mM, SDS 0.1%, Sodium fluoride 1 mM, EDTA 1 mM, Triton X-100 1%, 1X Protease inhibitor), resolved by SDS-PAGE, and transferred onto PVDF membranes. Primary antibodies for the *bone-fide* miR-155-5p target C/EBPβ (sc150, Santa Cruz Biotechnology) and normalizer Vinculin (#13901, Cell Signaling Tech) were detected by species-specific secondary HRP-linked antibodies, and revealed by using Pierce ECL 2 Substrate (Thermo Fisher Sc.). Image acquisition was performed on ChemiDoc XRS (Bio-Rad, Richmond, CA).

## Results

### Surrogate Molecular Classification of the CSS Cohort

Several genome-wide expression analyses have tried to identify clinically relevant molecular breast cancer subtypes (3–6). Among them, the PAM50 (4, 5) distinguishes five intrinsic subtypes: luminal A, luminal B, basal-like, HER2-enriched, and normal breast-like, characterized by different clinical behaviors. However, due to a lack of reimbursement, multigene tests are not readily available for all patients in many countries including Italy. Consequently, the use of immunohistochemistry (IHC) based biomarkers such as Ki-67 together with HER2 status has been proposed to distinguish ER-positive breast cancer cases at high risk of disease progression (i.e., *bona fide* Luminal B tumors) from those at low risk of disease progression (i.e., *bona fide* Luminal A tumors) (7). Instead, it is well established that *HER2*-amplification or intense overexpression (IHC 3+) is associated with worse prognosis in ER-positive BC patients. It is more difficult to classify the risk of ER-positive HER2-negative tumors because the prognostic performance of Ki-67 IHC staining shows a high variability among studies and laboratories (7). In our cohort, we evaluated two possible Ki-67 cut-offs: a 20% cut-off, as proposed by AIOM Guidelines (https://www.aiom.it/wp-content/uploads/2018/11/2018_LG_AIOM_Mammella.pdf), and the median cut off value of Ki-67 calculated in our study population (30%). As shown in Supplemental Table 3, only a Ki-67 cut-off of <30% was able to distinguish patients at low risk from patients at higher risk of disease progression (PFS HR = 0.38; 95%CI = 0.16–0.90; *P* = 0.029), metastases development (MFS HR = 0.34; 95%CI = 0.14–0.79; *P* = 0.012) and death (OS HR = 0.11; 95%CI = 0.01–0.87; *P* = 0.037) within the ER-positive subgroup. Thus, we defined Luminal A (LUMA) tumors as ER-positive/PgR positive, and HER2-negative with a low Ki-67 assessment (<30%), and Luminal B (LUMB) tumors as ER/PgR positive either HER2-positive or with a Ki-67 ≥ 30%. Overall, 94 cases (36%) were classified as Luminal A, 98 cases (37%) as Luminal B along with 34 HER2-amplified cases (13%), and 36 Triple Negative Breast Cancer cases (TNBC) (14%). The 21 remaining cases were not classified because HER2 or Ki-67 statuses were unknown.

### miR-155-5p Expression Is Associated With Unfavorable Prognostic Indicators in Both CSS Cohort and TCGA-BRCA Dataset

**Figure 1:**
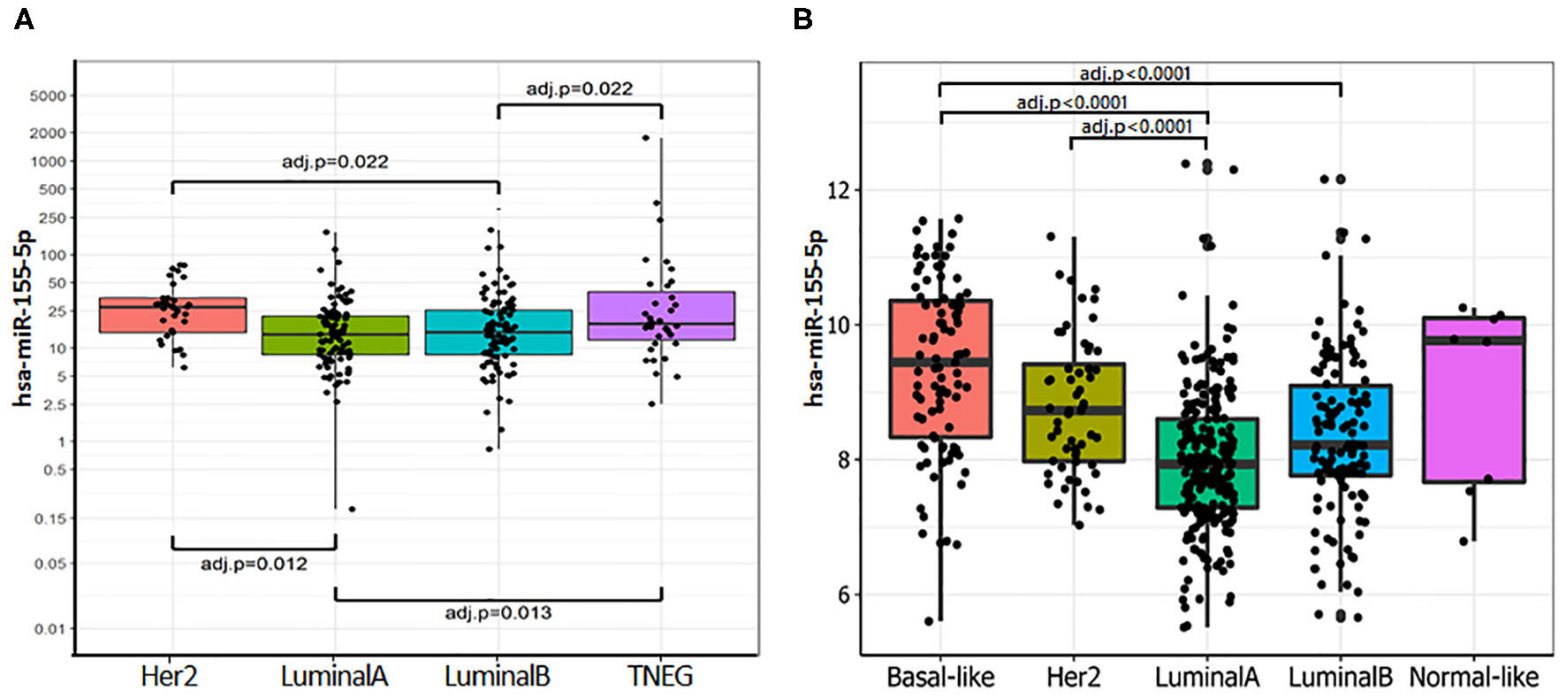
miR-155-5p is differentially expressed within the molecular breast cancer subgroups. **(A)** miR-155-5p expression within the surrogate molecular classification in CSS-cohort. High miR-155-5p expression was found in TNBC and HER2-amplified subgroups as compared with LUMA and LUMB tumors. **(B)** miR-155-5p expression within molecular subgroups identified by PAM50 in TCGA-BRCA dataset. High miR-155-5p levels were found in the Basal-like subgroup as compared with both Luminal A and Luminal B tumors, and in HER2-enriched subgroup as compared with Luminal A subgroup.

The expression of miR-155-5p [(hsa-miR-155-5p/RNU48) × 1,000] was evaluated in 283 primary breast tumors. As shown in Supplemental Table 4, miR-155 expression was associated with unfavorable prognostic indicators including high tumor grade (overall *P* = 0.0007; G2 vs. G3 *P* = 0.0053), reduced expression of ER (*r* = −0.243; *P* = 0.0002) and PgR (*r* = −0.240; *P* < 0.0001), and high Ki-67 expression (*r* = 0.215; *P* = 0.0005). Overall, these data suggest that miR-155 increased expression is associated with hormone receptor independency, high proliferation rate, and poor differentiation. However, no significant association was found with the pathological stage, lymph node status, and metastases development. These results were confirmed by the analysis of the TCGA-BRCA dataset (Supplemental Table 5). Then, we evaluated the expression of miR-155-5p across the surrogate molecular classification groups of the CSS cohort (*n* = 262) and the PAM50 subgroups for the TCGA-BRCA dataset (*n* = 505). In both cohorts, miR-155-5p was differentially expressed among the subgroups (CSS cohort *P* = 0.0008; TCGA-BRCA dataset *P* = 0.0005). In particular, higher miR-155 expression was found in HER2-amplified and TNBC subgroups, as compared with both Luminal A (*P* = 0.012 and 0.013, respectively) and Luminal B (both *P* = 0.0219) in the CSS cohort (Figure 1A). Accordingly, in the TCGA-BRCA dataset, the highest level of miR-155 was found in the basal-like subgroup followed by HER2-enriched tumors, whereas lower levels were detected in the Luminal A and Luminal B tumors. Pairwise comparison among groups evidenced statistically significant differences in the Basal-like subtype as compared with both Luminal A (*P* = 0.0001) and Luminal B subtypes (*P* = 0.0001), whereas the HER2-enriched subgroup showed significant differences only with the Luminal A subtype (*P* = 0.0001; Figure 1B). No significant associations were found between miR-155 expression and HER2 amplification/overexpression in neither CSS cohort nor TCGA-BRCA dataset.

When we sought to determine putative associations of miR-155 with clinical outcomes, no statistically significant associations between miR-155 levels and patients' survival emerged from univariable Cox Regression analyses, in either the total population or molecular subgroups of both CSS cohort and TCGA-BRCA dataset. Moreover, no association was found in the subgroups of CSS patients stratified according to therapeutic regimens (chemotherapy, radiotherapy, and anti-HER2 monoclonal antibodies; data not shown).

### miR-155-5p Is Differentially Expressed in Breast Cancer From TCGA-BRCA Dataset According to the Mutational Status of Homologous Recombination Genes

**Figure 2:**
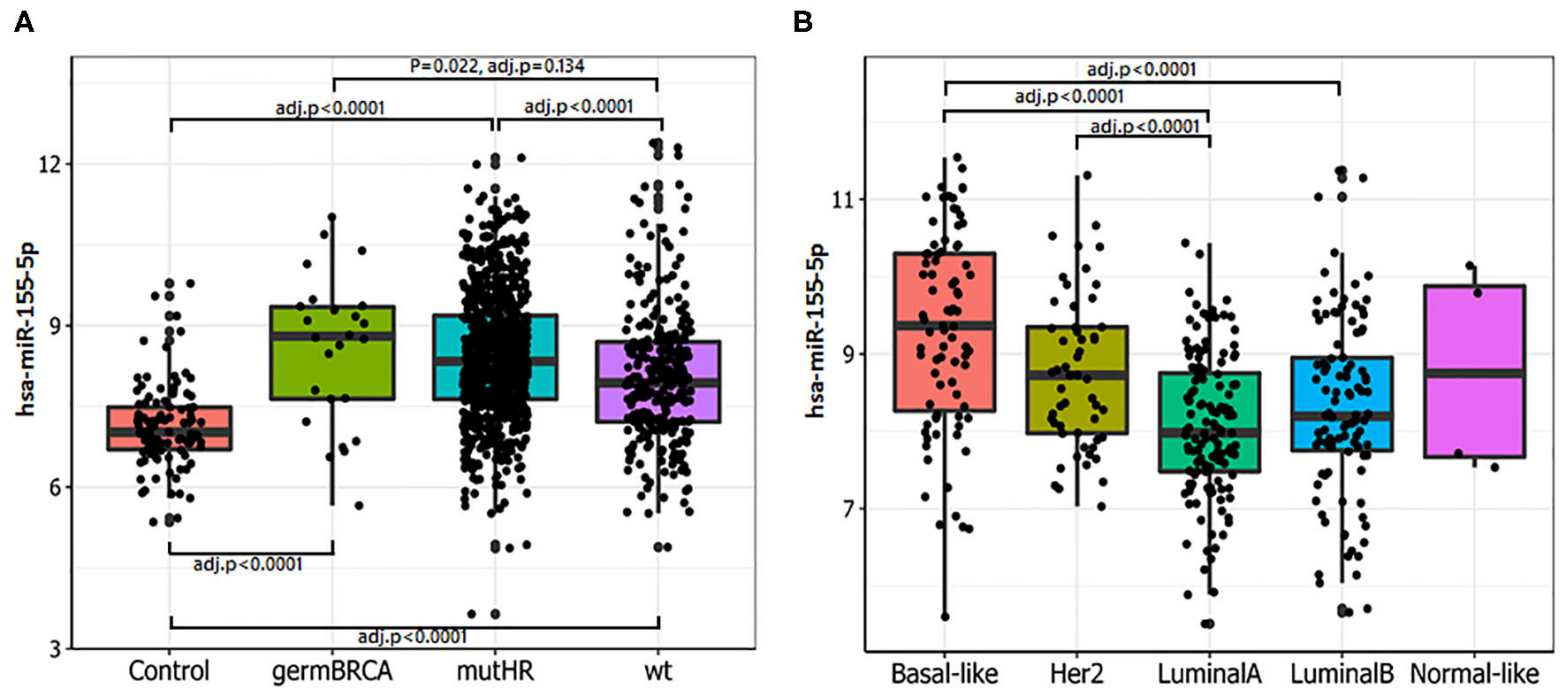
miR-155-5p expression is associated with mutations in HR genes and basal-like phenotype. **(A)** Higher miR-155-5p levels were detected in tumors carrying mutations in HR genes. Statistically significant differences were found among controls with either germline *BRCA1/2* mutated tumors (germ*BRCA*), HR mutated (mutHR) or non HR mutated tumors (wt). High miR-155-5p expression was found in both germ*BRCA* and mutHR tumors as compared with the wild type (wt) subgroups. **(B)** In mutHR tumors, miR-155-5p is differentially expressed according to PAM50 classification. Higher miR-155-5p levels were found in Basal-like and HER2 tumors as compared with the Luminal A subtype, and in the HER2-amplified tumors as compared with Luminal A subtype.

miR-155-5p has been found to be epigenetically repressed by wild type BRCA1 through its association with HDAC2 at *MIR155* gene promoter and such a regulation is impaired by the R1699Q variant of *BRCA1* (32). This, together with the findings that high levels of miR-155-5p associate with the Basal Like phenotype, a common feature of *BRCA-* mutated tumors, spurred us to investigate whether miR-155-5p may be associated with Homologous Recombination Deficiency (HRD), and thus represents a typical trait of *BRCAness*. To test this hypothesis, we selected those breast cancer cases for which the mutational status of 24 genes involved in the Homologous recombination pathway (Table 1) was available (*n* = 1,095 plus 104 normal controls) from the TCGA-BRCA dataset. Of those tumors, 801 carried at least one mutation (indel, missense or CNV) in one of the homologous recombination genes. Information about the expression levels of miR155-5p was available for 793 out of 801 mutated tumors and 291 out of the 295 cases without mutation in these genes (*wt*). Of the 793 mutated cases, 26 patients carried germline mutations in *BRCA1* or *BRCA2* (germMut), while 763 carried somatic mutations in homologous recombination genes (mutHR). Overall, 3,144 mutations were reported of which 91% were CNV and 9% were SNP or indels (Supplemental Table 6). The list of SNPs and indel mutations, together with their putative functional relevance, is reported in Supplemental Table 7. As shown in Figure 2A, miR-155 was increased in tumor samples (Median 302.94, IQR = 184.62–536.70) as compared with controls (*n* = 104) (Median = 129.96, IQR = 103.57–179.24; *P* = 0.0001). Higher miR-155 expression was found in the mutHR subgroup (Median = 446.600, IQR = 198.82–596.07) as compared with the *wt*HR subgroup (Median = 246.55, IQR = 148.43–419.62; *P* < 0.0001). The fact that no statistically significant differences were found for the germ*BRCA* subgroup (Median = 446.60, IQR = 186.68–656.97) as compared with both *wt* (*P* = 0.134) and *mut*HR (*P* = 1.00), might be ascribed to the low number of germMut cases (*n* = 26; Figure 2A). Furthermore, the distribution of PAM50 subtypes differed significantly among the groups, with Basal-like and HER2-amplified tumors being more frequent in *mut*HR (83 and 55, respectively) as compared with *wt* (8 and 1, respectively; *P* = 0.0001). In the *mut*HR subgroup (Figure 2B), miR-155 was differentially expressed among the intrinsic molecular subtypes with higher expression in the Basal-Like (Median = 660.07, IQR = 296.79–1,262.98) and HER2-enriched (Median = 423.58, IQR = 251.10–653.04) tumors as compared with Luminal subtypes (LUMA Median = 252.87, IQR = 178.77–435.31, and LUMB Median = 292.89, IQR = 211.76–511.78; *P* < 0.0001; Figure 1B). Overall, these data indicate that miR-155 expression may represent a hallmark of homologous recombination deficiency (HRD).

**Table 1:** Summary of mutations detected in the 24 homologous recombination genes evaluated in the TCGA-BRCA dataset.

**Gene symbol**	**Ensembl ID**	**Cytoband**	**Copy Number Variation**		**Frameshift mutation**		**SNP**		
			**Deletions**	**Amplifications**	**Deletion**	**Insertion**	**Missense**	**Non sense**	**Splice site**
ARID1A	ENSG00000117713	1p36.11	172	19	10	3	13	14	3
ATM	ENSG00000149311	11q22.3	157	34	1	0	15	5	2
ATRX	ENSG00000085224	Xq21.1	21	18	2	0	39	3	1
BAP1	ENSG00000163930	3p21.1	140	11	1	1	9	1	0
BARD1	ENSG00000138376	2q35	47	36	0	0	0	0	0
BLM	ENSG00000197299	15q26.1	14	92	1	0	15	1	0
BRCA1	ENSG00000012048	17q21.31	89	40	4	0	12	5	2
BRCA2	ENSG00000139618	13q13.1	86	73	5	0	18	3	1
BRIP1	ENSG00000136492	17q23.2	11	233	0	1	9	1	1
CHEK1	ENSG00000149554	11q24.2	157	25	0	0	2	0	0
CHEK2	ENSG00000183765	22q12.1	24	39	0	0	6	1	0
FANCA	ENSG00000187741	16q24.3	64	40	0	0	8	0	0
FANCD2	ENSG00000144554	3p25.3	25	97	0	1	8	2	0
FANCE	ENSG00000112039	6p21.31	31	66	0	0	3	0	0
FANCF	ENSG00000183161	11p14.3	24	56	0	1	2	0	0
FANCG	ENSG00000221829	9p13.3	18	56	0	0	3	0	0
FANCL	ENSG00000115392	2p16.1	7	59	0	0	1	0	1
MRE11	ENSG00000020922	11q21	89	54	0	0	0	0	0
NBN	ENSG00000104320	8q21.3	19	150	0	0	5	1	0
PALB2	ENSG00000083093	16p12.2	12	42	0	0	5	1	0
RAD50	ENSG00000113522	5q31.1	37	31	0	1	8	1	0
RAD51	ENSG00000051180	15q15.1	100	21	0	0	2	0	0
RAD51B	ENSG00000182185	14q24.1	89	52	0	0	2	0	1
WRN	ENSG00000165392	8p12	51	39	1	0	8	0	0

### miR-155-5p May Indirectly Affect Cell Response to PARP-1 Inhibitor Olaparib

PARP-1 inhibitor Olaparib has been initially approved as monotherapy for the maintenance treatment of adult patients with platinum-sensitive relapsed *BRCA-*mutated (germline or somatic) high-grade ovarian cancer (33). More recently, results from the OlympiAD Phase 3 clinical trial indicate that Olaparib is also effective in *BRCA1* or *BRCA2* mutated HER2-negative metastatic breast cancer (34, 35). Our findings that high levels of miR-155-5p associate with the presence of mutations at HR genes prompted us to investigate whether miR-155-5p may affect the response to the PARP inhibitor Olaparib to some extent.

**Figure 3:**
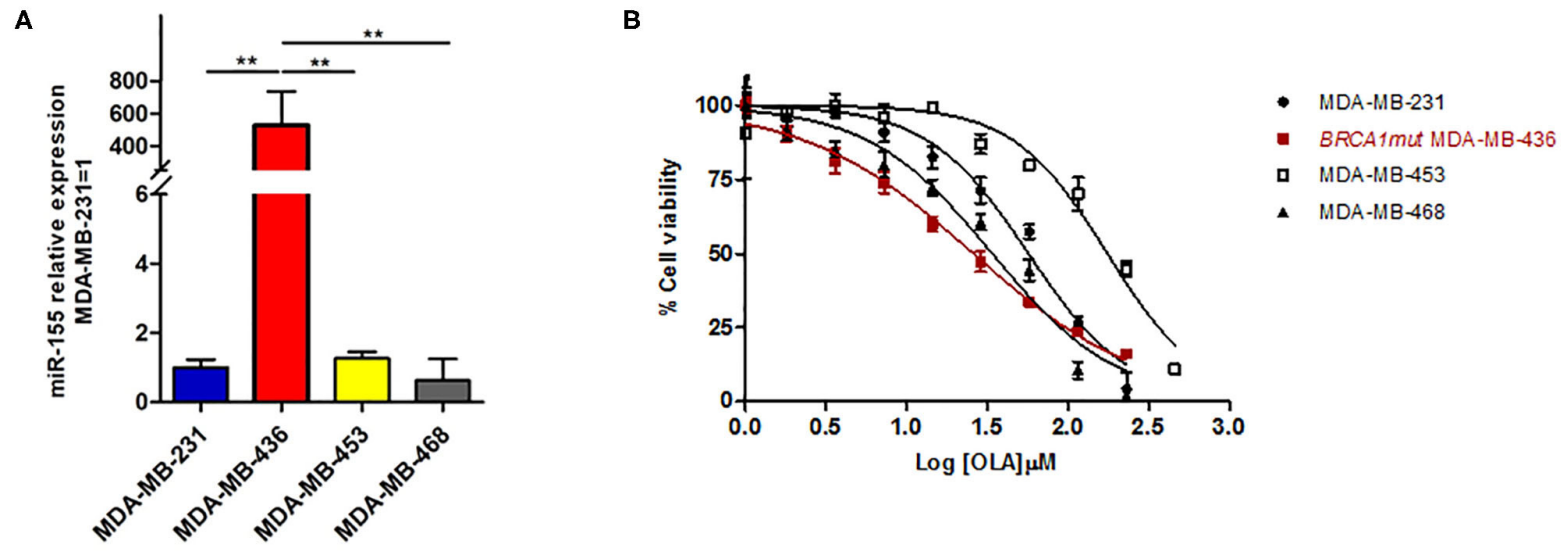
**(A)** miR-155-5p endogenous expression levels are significantly increased in the *BRCA1*mut MDA-MB-436 cell line as compared to the other wt*BRCA*-TNBC cell lines. miR-155-5p was quantified by RT-qPCR and normalized using RNU48 as endogenous control. Data are presented as fold increase over the expression levels of MDA-MB-231 and were derived from 6 biological replicates (Student's t-test, **p < 0.01). **(B)** Olaparib similarly affects cell viability of basal-like TNBC cell lines independently of *BRCA-*status. Growth curves of MDA-MB-231, MDA-MB-436, MDA-MB-468, and MDA-MB-453 (TNBC cell lines) were construed following 72 h of treatment with a range of 2-fold serial dilutions of Olaparib compound (230–1.8 μM) and cell viability assessment by PrestoBlue^TM^ Reagent. The percentage of viable cells was plotted against Log-transformed Olaparib concentrations. Data are presented as mean (±SD) of three independent experiments.

**Figure 4:**
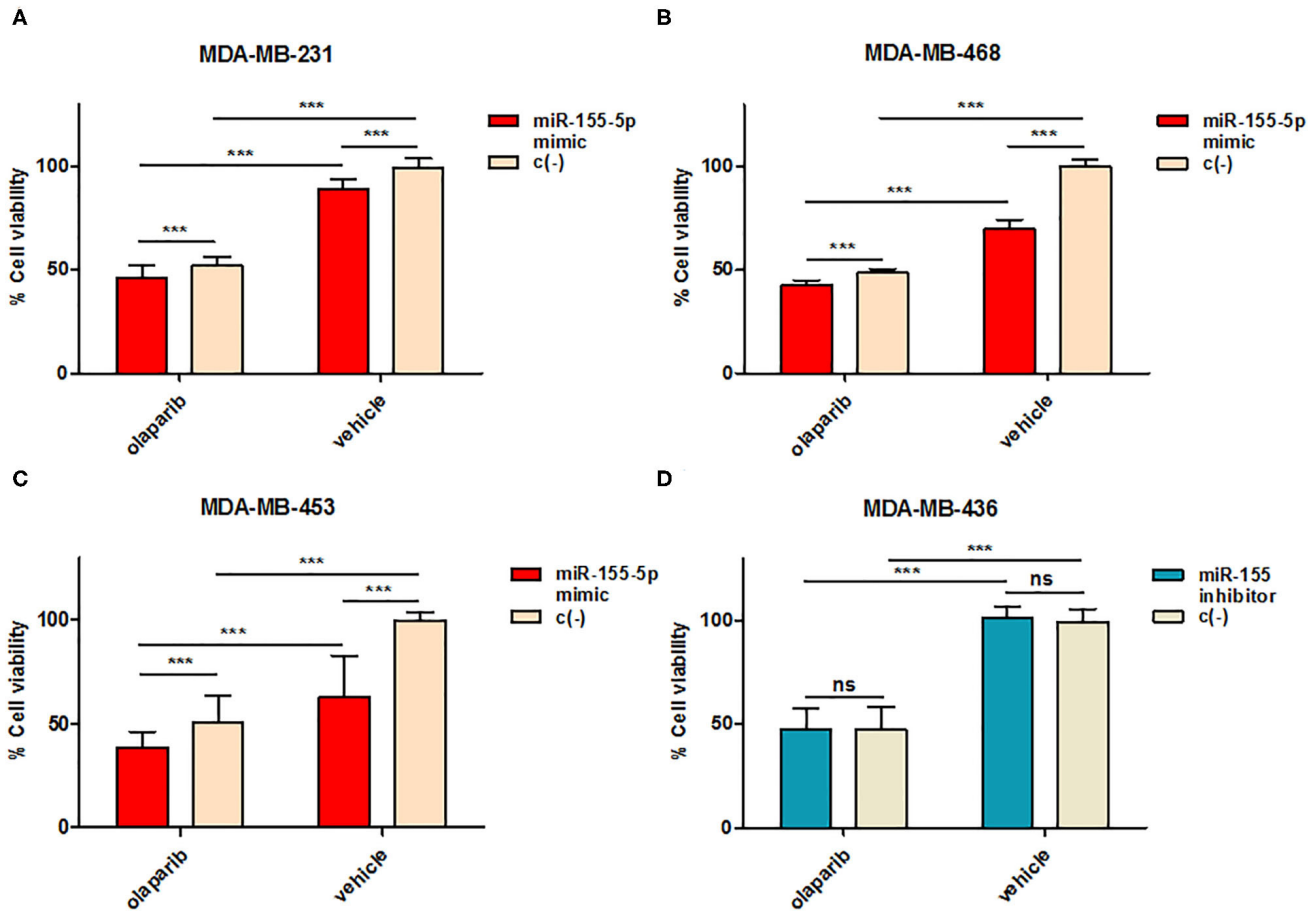
miR-155-5p effects on cell sensitivity to Olaparib. Cell viability was assessed by PrestoBlue^TM^ reagent after induced overexpression in **(A)** MDA-MB-231, **(B)** MDA-MB-468, and **(C)** MDA-MB-453 or inhibition in **(D)** MDA-MB-436 of miR-155-5p by mirVana miR-155-5p mimic or inhibitor followed by Olaparib administration for 72 h. Data are presented as percentage of viable cells calculated in each condition (Olaparib vs. Vehicle) with respect to vehicle-treated c(–)-transfected cells, and represent the mean (±SD) of four independent experiments (Student's t-test, ***p < 0.001, ns = not significant).

**Figure 5:**
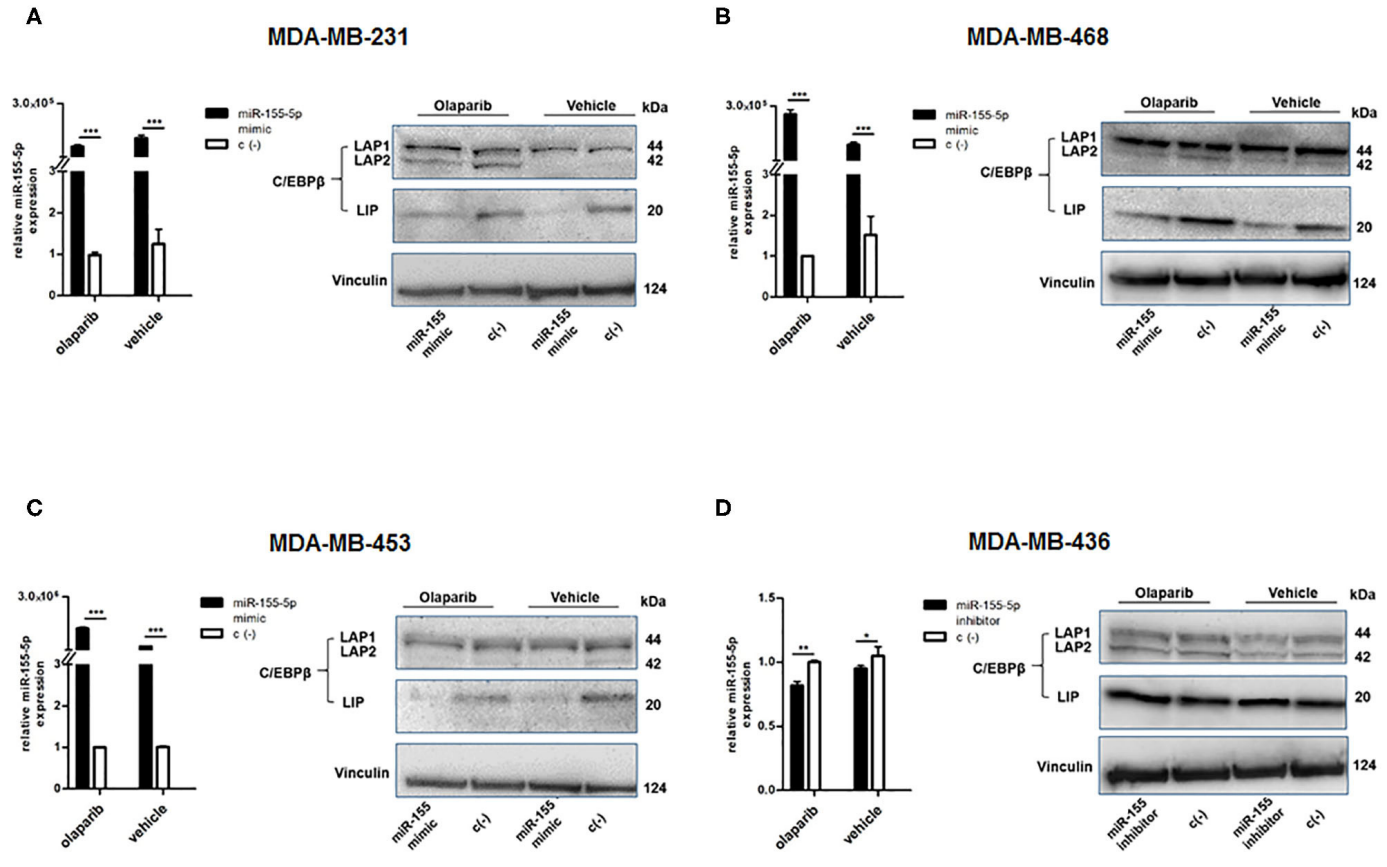
Evaluation of efficacy and functionality of miR-155-5p overexpression/inhibition. miR-155-5p expression levels and western blot analyses of C/EBPβ protein levels in **(A)** MDA-MB-231, **(B)** MDA-MB-468, **(C)** MDA-MB-453, and **(D)** MDA-MB-436. miR-155-5p expression levels are presented as fold increase (2^−ΔΔCT^) with respect to correspondent negative control c(–)-transfected cells, in the presence of Olaparib or vehicle, and represent the mean (±SD) of four independent experiments (Student's t-test, *p < 0.05, **p < 0.01; ***p < 0.001).

To this aim, we selected four breast cancer cell lines of TNBC, including the *BRCA1* mutant cell line MDA-MB-436 (basal-like), and the *BRCA1* wild type lines with *BRCA1* allelic loss but normal transcript levels MDA-MB-468 (basal-like), MDA-MB-231 (claudin-low) and MDA-MB-453 (LAR-subtype). We did find that miR-155-5p endogenous levels were significantly elevated in mut*BRCA1* MDA-MB-436 cells compared to the other cell lines (Figure 3A), suggesting the role of this *BRCA1* pathogenic variant in abrogating negative regulation of miR-155-5p (30, 32). Interestingly, we also observed that, when we tested a wide range (see section Materials and Methods) of Olaparib concentrations that reduced cell viability significantly in a short time-interval of 72 h (IC50_72_), both basal-like TNBC cell lines encountered a 50% reduction of viability upon Olaparib treatment at similar doses independently of *BRCA1* status (MDA-MB-436, 25.80 μM, 95%CI = 24.52–27.15; MDA-MB-468 33.81 μM 95%CI = 31.24–36.60) (Figure 3B). Next, to test the hypothesis that miR-155-5p might affect response to Olaparib, we ectopically overexpressed miR-155-5p in the less-responsive *wt BRCA1* cell lines (MDA-MB-453, MDA-MB-231, and MDA-MB-468), and treated them at correspondent IC50_72h_, with the exception of MDA-MB-453 cell line, for which we chose a lower Olaparib dose (i.e., IC40_72 h_, 60 μM) because showing reduced tolerance to transfection procedures compared to the other cell lines. The ectopic overexpression of miR-155-5p itself induced a very significant decrease in cell viability in all cell lines compared to control (c(–)) cells. Moreover, when we treated both miR-155-5p overexpressing and control cells with Olaparib compound, we did observe a further reduction of cell viability, indicating that Olaparib and miR-155-5p may cooperate, and severely impair the survival of cancer cells (Figures 4A–C). Nevertheless, no alterations in Olaparib cell sensitivity were observed upon miR-155-5p inhibition in MDA-MB-436 (Figure 4D), suggesting that miR-155-5p regulatory role may be necessary but not sufficient for determining Olaparib sensitivity. The efficacy, as well as functionality of miR-155-5p overexpression/inhibition, was checked by both RT-qPCR and western blot analyses of its *bone-fide* target C/EBPβ protein levels (Figures 5A–D, Supplemental Figure 1).

### Alterations of C/EBPβ Isoforms Abundance May Mediate the Growth Inhibitory Effect of miR-155-5p Coupled With Olaparib

As mentioned above, we evaluated the protein levels of miR-155-5p *bona-fide* target C/EBPβ (36) by western blot to ensure that ectopic modulation of miR-155-5p endogenous levels in the transfected-cell lines was functional. C/EBPβ (CCAAT/enhancer-binding protein) is a well-known transcription factor playing a central role in controlling the growth and differentiation of normal mammary gland, and it is produced in three isoforms through alternative usage of different start codons: the transcriptional activators liver activating protein 1 and 2 (LAP1 and LAP2), and the transcriptional inhibitor LIP, which can inhibit LAPs-mediated gene activation (36). The analyses of C/EBPβ protein levels in our cell lines showed a decreased level of the LIP isoform in miR-155-5p overexpressing cells compared to control cells, in both Olaparib- and vehicle-treated cells (Figures 5A–C), demonstrating the functionality of enforced miR-155-5p overexpression, and likely explaining the decrease in cell viability we observed after miR-155-5p overexpression. More interestingly, we noticed that, together with the miR-155-5p-mediated LIP reduction, a marked increase in LAP2 isoform in the *claudin-low* MDA-MB-231 cell line was induced specifically by Olaparib administration (Figure 5A). Since the smaller LAP2 isoform is considered to be the most transcriptionally active of the C/EBPβ isoforms, it is likely that its overexpression coupled with LIP downregulation, resulting in a LIP:LAP imbalance, severely impairs cell proliferation. We could not appreciate significant differences in MDA-MB-436 cell line, although transfection appeared to be successful, probably due to high C/EBPβ basal levels in this cell line, as shown by negative control (c(–))-transfected cells (Figure 5D).

## Discussion

miR-155-5p is one of the best conserved and multifunctional miRNA involved in several physiological processes, such as proliferation, cell cycle, apoptosis, and differentiation (8–12). Altered expression of miR-155-5p has been found to be associated with hematopoietic lineage differentiation, immune response, inflammation, and tumorigenesis (8–12). Several studies suggest a role of miR-155-5p in breast cancer development and progression. However, its putative role as a biomarker is still controversial, likely due to limited patient sample sizes and discrepancies among studies in terms of methodologies and experimental models (13–22).

The primary aim of our study was the evaluation of miR-155-5p expression in a large cohort of breast cancer cases with a median follow up of 81 months. Our results were then validated in the TCGA-BRCA dataset. Consistently with previous reports, miR-155-5p expression was associated with negative prognostic factors including reduced expression of hormone receptor, high histological grade, and proliferation index measured by Ki67 expression in both our cohort and TCGA-BRCA dataset. Indeed, when we evaluated miR-155-5p expression within the subgroups identified by the surrogate molecular classification in the CSS cohort, and the intrinsic molecular classification in TCGA-BRCA dataset, the highest levels of expression were found in the TNBC and Basal-like subgroups followed by HER2-amplified tumors and the lowest expression in the Luminal subgroups. Overall, these data support the oncogenic role of miR-155-5p in deregulating cell proliferation and differentiation within the hormone-independent breast cancer subtypes. No associations were found with lymph node status and metastases development (Supplemental Tables 4, 5), as well as miR-155-5p did not correlate with patient's survival in univariable Cox regression analyses in both cohorts. These data rule against the role of miR155 as prognostic biomarkers in breast cancer. Nevertheless, the small number of TNBC (*n* = 34) and HER2 (*n* = 36) cases in our cohort, and the absence of information about PFS and MFS in the publicly available TCGA-BRCA dataset cannot completely exclude such a role in these breast cancer subgroups. Thus, this result should be further evaluated in retrospective or prospective studies with a specific focus on these subgroups.

Breast cancers with loss-of-function mutations in *BRCA1* or *BRCA2* genes are deficient in the HR (Homologous Recombination) pathway that manages the repair of DNA DSBs (Double Strand Breaks); hence, they are exquisitely sensitive to poly(ADP ribose) polymerase (PARP) inhibitors (37). This sensitivity to PARP inhibition derives from the synthetic lethality of cells with defective homologous recombination-mediated DNA repair to toxic replication intermediates generated on chromatin by PARP “trapping” (38). Preclinical studies and clinical trials in breast, ovarian cancer, and other cancers have shown the PARP inhibitors efficacy in *BRCA1-* and *BRCA2*-mutant patients (38–40). This led in 2014 to the approval by drug regulatory agencies of the PARP inhibitor Olaparib for the treatment of patients with recurrent ovarian cancer and *BRCAs* mutations (41). Encouraging results with Olaparib have also been obtained in patients with metastatic breast cancer-bearing germline *BRCAs* mutations (40–42). The randomized, phase 3 OlympiAD trial showed that among germline BRCA mutated patients affected by ER-positive HER2-negative metastatic TNBC, median progression-free survival was significantly longer with oral Olaparib monotherapy than with standard chemotherapy (34). This has finally led to Olaparib FDA-approval in January 2018 for g*BRCA1/2*+ HER2–breast cancers in the metastatic setting (35).

Although germline *BRCA* (germ*BRCA*) mutations still remain the best clinical biomarkers for response to PARP inhibitor therapy (34, 43), genetic and epigenetic inactivation of other components of the homologous recombination apparatus can lead to HRD in sporadic cancers, broadly termed BRCAness (44). These alterations can occur in the form of germline mutations in HRR-associated genes, such as *PALB2, FANCM, CHECK2*, and *RAD51C/D* (44) or somatic mutations including *ATM, BAP1, CDK12* (44) so that numerous studies are currently testing the possibility to apply mutational signatures to the prediction of PARP-inhibitors responsiveness (45, 46). BRCAness and HRD also involve methylation of the *BRCA*-promoter, which appears to be most common in TNBCs (47, 48). Breast tumors with *BRCA1* methylation also show the higher histological grade, like that of *BRCA1-*mutated tumors (49). Additional contributors to HRD involve copy number alterations (CNA), and early research has revealed that the basal-like subtype of breast cancer has higher numbers of gains/losses, while the Luminal B subtype has a more frequent high-level DNA amplification (50).

Thus far, a few miRNAs have emerged as determining the BRCAness phenotype and, therefore, the response to PARP inhibition-based therapy (51, 52). miR-155-5p has been previously found to increase the tumor mutation load and promote the so-called “mutator phenotype” in inflammation-driven tumors due to the combined targeting of cell-cycle regulators and DNA repair enzymes (25, 26). Moreover, miR-155-5p expression has been found to be epigenetically controlled by BRCA1 through the recruitment of histone deacetylase 2 (HDAC2) at the *MIR155* gene promoter (32) that ultimately leads to transcriptional silencing. In human cell lines, *BRCA1*-deficient cells showed 50-fold higher miR-155-5p levels compared with those with functional BRCA1. Finally, the transient overexpression of BRCA1 protein reduces the expression of miR-155-5p and, in clinical samples, miR-155-5p levels were 2- to 6-fold higher in *BRCA1*-mutant tumors compared to wt *BRCA1* tumors.

For these reasons, we wondered whether miR-155-5p might correlate with defects in HR genes and represent a putative trait of BRCAness in the breast cancer of Triple Negative subtype (TNBC). Overall, we found that defects in the homologous recombination genes are a common feature of breast cancer, with 73% of cases in the TCGA-BRCA dataset carrying at least one germline or somatic mutation in HR genes. Almost all Basal Like and HER2-associated tumors were HR mutated. When we tested the putative association of miR-155-5p with the presence of mutations in Homologous Recombination genes, we were not surprised to find that miR-155-5p did show higher expression levels in the mutHR subgroup rather than in the wtHR subgroups in the TCGA Breast cancer dataset.

Next, we assessed the capability of miR-155-5p to alter the response to the first-in-class PARP inhibitor Olaparib (AZD2281) of four BC cell lines, differing in both molecular subtype and *BRCA1* status. As expected, the MDA-MB-436 cells, representing a *BRCA1*-mutant basal-like TNBC cell line, showed a higher sensitivity to a short-term (72 h) treatment with Olaparib alone (IC50_72h_ 25.80 μM, 95%CI = 24.52–27.15) compared to the other cell lines. In addition, we also observed that, independently of *BRCA*-mutational status, the second basal-like TNBC cell line MDA-MB-468, carrying normal levels of BRCA1 transcript despite allelic loss, and low expression of ATM, did show a high sensitivity to Olaparib (IC50_72h_ 33.81 μM, 95%CI = 31.24–36.60), consistent with previous findings supporting this cell's *BRCA*ness phenotype (49), and confirming that HR deficiency might be exploited to get a cancer cell response beyond *BRCA-*mutations. Then, when we enforced miR-155-5p overexpression in MDA-MB-231, MDA-MB-468, and MDA-MB-453 cell lines, we observed a significant decrease in cell viability following miR-155-5p overexpression alone in all cell lines. Interestingly, miR-155-5p-induced overexpression, followed by Olaparib exposure, resulted in an increased sensitivity to PARP inhibition and a further reduction in cell viability.

In order to determine the functionality of modulated miR-155-5p in our *in vitro* models, we analysed the protein expression levels of the transcriptional factor C/EBPβ, known to be one of *bone-fide* targets of miR-155-5p that plays an important role in regulating the balance between cell differentiation and proliferation in the mammary gland, according to the differential expression pattern of its isoforms. In particular, C/EBPβ has been recognized as core factor of the TGFβ cytostatic program, being essential for the induction of p15INK4b and the repression of c-MYC (53), and mediator of Ras-induced senescence (54). The association of C/EBPβ defects with breast cancer progression has been mostly attributed to aberrant levels of LIP isoform that, once elevated, may antagonize the transcriptional activities of LAPs and thus confer a growth advantage to cancer cells (55–58). Consistently with these data, ectopic overexpression of miR-155-5p in our cell lines showed to reduce the levels of the inhibitory LIP isoform in miR-155-5p-overexpressing cells with respect to control-transfected cells in both Olaparib- and vehicle-treated cells, thus demonstrating the functionality of synthetic oligos, and likely explaining the decrease in cell viability we observed after miR-155-5p mimic transfection (Figures 5A–C). Interestingly, we could detect a significant increase in the C/EBPβ LAP2 isoform protein level as a specific pattern of Olaparib treatment compared to vehicle in *claudin-low* MDA-MB-231 cells (Figure 5A). We might speculate that the decreased cell viability achieved in this cell line by miR-155-5p overexpression and Olaparib combination was a result of an enforced cell growth inhibitory effect exerted by LAP2 regulated genes and PARP inhibition. Moreover, since increased LAP2 was observed specifically in Olaparib-treated cells, we cannot rule out that the specific Olaparib action may go beyond PARP enzymes inhibition, and stimulate additional pathways toward growth inhibition. Hence, these data raise the question of whether increasing instead of inhibiting miR-155-5p in different cancer settings may enhance PARPi efficacy. Indeed, previous reports have already demonstrated the capability of miR-155-5p of boosting the anticancer immune response by targeting anti-inflammatory factors and immune checkpoints, including CTLA4 and PD-L1 [reviewed in (59)], something that may be of relevance for putative PARPi and Immunocheckpoints Inhibitors (ICI) combined therapies (60).

However, our results as well as the *in vitro* evidence that inhibition of miR-155-5p in *BRCA1*mut MDA-MB-436 cell line did not affect the higher sensitivity to PARP inhibition support the idea that several mechanisms underlie sensitivity to PARPi, and the understanding of the BRCAness as well as considering the specific BC subtype context warrants further investigation.

In conclusion, the analysis of two independent breast cancer cohorts corroborates the oncogenic role for miR-155-5p. Indeed, increased miR-155-5p expression was associated with loss of hormone receptor, reduced differentiation, and high proliferation rate. Consistently, miR-155-5p was differentially expressed in breast cancer molecular subgroups, with the highest expression in basal-like and HER2-related tumors. In addition, higher miR-155-5p expression was found in breast cancer cases from the TCGA dataset carrying mutations in HR genes. Lastly, we propose that a novel mechanism of synthetic lethality mediated by PARP inhibition and miR-155-5p may promote cancer cell death, and suggest miR-155-5p as an additional trait of the BRCAness phenotype.

Currently, several scoring systems aimed to identify HRD tumors are under investigation. These systems showed some predictive value in metastatic breast cancer patients treated with platinum derivatives (61). By using a high-depth whole-genome sequencing approach, Davies H. et al. developed a tool, they called HRDetect, as a predictor of *BRCA1* and *BRCA2* deficiency based on mutational signatures (62). Such a technology was able to identify six different mutational signatures and classify *BRCA1/2-*deficient tumors correctly with 98.7% sensitivity. In addition, in a cohort of 560 individuals with BC, HRDetect identified 22 tumors with somatic loss-of-*BRCA1* or *BRCA2* and 47 tumors with functional BRCA1/2 deficiency, none of which had mutations detected with standard analysis (61). However, to date, HRDetect has not been correlated yet with therapeutic responses to PARPi. Another recent study evaluated a novel gene expression signature-generating algorithm to predict therapeutic response to PARPi (63). Our results indicate that, together with mutation and gene expression signatures, miRNA expression analysis may aid in evaluating the competency of homologous recombination, and thus eventually increase the ability to predict response to PARPi, in order to ultimately identify additional breast cancer patients eligible for PARP-inhibition including regimens.

## Data Availability Statement

The datasets presented in this study can be found in online repositories. The names of the repository/repositories and accession number(s) can be found in the article/Supplementary Material.

## Ethics Statement

The studies involving human participants were reviewed and approved by Fondazione IRCCS Casa Sollievo della Sofferenza. The patients/participants provided their written informed consent to participate in this study.

## Author Contributions

BP, RB, and PP: substantial contribution to conception and design of the study. BP, RB, MD, MR, SB, and SR: performed analytical procedures. AF and MC: performed statistical analyses. TB and TM: performed bioinformatics analyses. VV, MM, EM, and RM: collected clinical follow up data and reviewed the manuscript. PG: pathological evaluation of specimens. PP, BP, RB, MD, AF, MC, TB, and TM: analysis and interpretation of data. PP, BP, RB, AF, MC, and TM: manuscript writing. VF and ME: critical review of the manuscript. All authors: reviewed the manuscript.

## Conflict of Interest

The authors declare that the research was conducted in the absence of any commercial or financial relationships that could be construed as a potential conflict of interest.

## Supplementary Material

Supplemental Figure 1 miR-155-5p expression levels following enforced overexpression/inhibition of miR-155-5p. miR-155-5p levels were quantified by RT-qPCR. Relative expression levels of miR-155-5p mimic/inhibitor-transfected cells were normalized to correspondent negative control c(–)-transfected cells (2^−ΔΔCT^) to rule out effects due to transfection procedures. Normalized data are presented as fold increase over the expression levels of MDA-MB-231 and represent the mean (±SD) of four independent experiments.

Supplemental Table 1 Clinicopathological characteristics of the CSS patient cohort (*n* = 283).

Supplemental Table 2 Clinicopathological characteristics of the TCGA-BRCA dataset (*n* = 1,095).

Supplemental Table 3 Univariable Cox Regression survival analysis evaluating two different KI-67 cut-off to estimate di risk of disease progression, metastases development, and death in ER positive breast cancer patients.

Supplemental Table 4 Associations between tumor clinicopathological characteristics and miR-155-5p expression in the CSS-cohort.

Supplemental Table 5 Associations between tumor clinicopathological characteristics and miR-155-5p expression in the TCGA-BRCA dataset.

Supplemental Table 6 Number of SNPs and indel detected according to gene and variant type.

Supplemental Table 7 List of SNPs and indel mutation detected in homologous recombination genes and their putative functional consequences.
