# Supplementary figures and images for: Hsa-miR-155-5p Up-Regulation in Breast Cancer and Its Relevance for Treatment With Poly[ADP-Ribose] Polymerase 1 (PARP-1) Inhibitors
Source: Front Oncol. 2020 Aug 12;10:1415. doi: 10.3389/fonc.2020.01415 (PMC7435065; doi:10.3389/fonc.2020.01415)

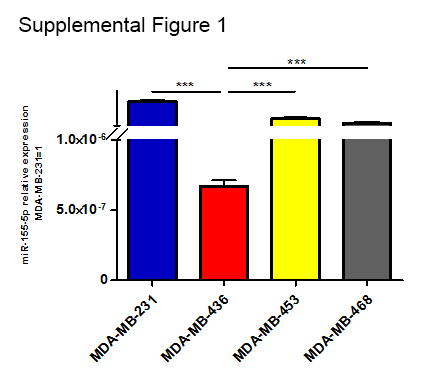

Supplement: Supplemental Figure 1 — miR-155-5p expression levels following enforced overexpression/inhibition of miR-155-5p. miR-155-5p levels were quantified by RT-qPCR. Relative expression levels of miR-155-5p mimic/inhibitor-transfected cells were normalized to correspondent negative control c(–)-transfected cells (2−ΔΔCT) to rule out effects due to transfection procedures. Normalized data are presented as fold increase over the expression levels of MDA-MB-231 and represent the mean (±SD) of four independent experiments. [file Image_1.TIF]
